# Special Issue—Resistant Staphylococci in Animals

**DOI:** 10.3390/vetsci10040240

**Published:** 2023-03-23

**Authors:** Bryan K. Markey, Finola C. Leonard

**Affiliations:** Section of Veterinary Pathobiology, School of Veterinary Medicine, University College Dublin, Belfield, D04 W6F6 Dublin, Ireland

Staphylococci figure prominently among those bacteria demonstrating antimicrobial resistance (AMR) and are thus responsible for significant problems concerning the treatment of the animals and humans that they infect. In particular, livestock-associated methicillin-resistant *Staphylococcus aureus* (LA-MRSA) and methicillin-resistant *Staphylococcus pseudintermedius* (MRSP) are of growing concern. *Staphylococcus schleiferi* and coagulase-negative staphylococci occur as commensals of the skin and mucous membranes of animals. However, they have been implicated in a variety of infections and are frequently resistant to one or more antimicrobial classes. This Special Issue assembles a collection of original articles that shed further light on these fascinating bacteria and their potential impacts on both veterinary medicine and public health.

The review by Lynch and Helbig [1] provides a timely update of our knowledge regarding *Staphylococcus pseudintermedius*, the most common pathogen isolated from skin disease samples (particularly pyoderma) from dogs [2]. The high prevalence of methicillin-resistance and associated resistance to many other antimicrobials in isolates of *S. pseudintermedius* constitutes a growing concern. A range of novel potential treatments including vaccination and phage therapy are discussed.

Chanayat et al. [3] investigated the staphylococcal cassette chromosome *mec* (SCC*mec*) type and the antimicrobial susceptibility of staphylococci isolated from superficial pyoderma infections affecting dogs in Thailand. SCC*mec* type V was found in *S. aureus*, the *S. intermedius* group, *S. lentus*, *S. xylosus,* and *S. arlettae*, and although the authors do not state whether the coagulase-negative species were considered the primary bacterial pathogens in the cases from which they were isolated, the need to reduce environmental contamination and educate veterinary personnel and clients about the potential for the transmission to and from dogs of all resistant staphylococci is emphasized. The need for hygiene is supported by the results of another recent longitudinal study of MRSP-infected dogs conducted by Frosini et al. [4], in which it was reported that 45% of households were contaminated with MRSP. The data presented by Chanayat et al. [3], includes two *S. pseudintermedius* isolates that showed MICs < 2 µg/ml, an interesting finding in light of the publication by Wegener et al. [5] suggesting that such isolates showing low-level resistance may be susceptible to treatment with higher doses of beta-lactams or more frequent administration.

As well as characterizing *S. pseudintermedius* isolates causing superficial pyoderma in Taiwanese dogs, Lai et al. [6] also examined the risk factors that might lead to owners acquiring *S. pseudintermedius* from their pets. Although no significant association was found, high odds ratios were obtained for “keeping three or more dogs” and “dogs can lick the owner’s face”, suggesting support for recent publications describing the potential for *S. pseudintermedius* infections in human hosts [7,8].

Certain foods and food production systems may present a pathway for the transmission of MRSA to humans. Benrabia et al. [9] detected MRSA in 30% of poultry farms in Algeria and provided a reasonable argument for conducting interventions to reduce spread between farms and mitigate the contamination of the food chain. The lack of the molecular typing of isolates was a limitation of this study, as it largely precluded source attribution and comparison with MRSA isolates reported worldwide. Nevertheless, the documentation of MRSA isolation rates in food-producing animal species in different geographical regions is essential to obtain a comprehensive understanding of this public health challenge.

*Staphylococcus aureus* is still the leading cause of bovine mastitis in many countries. Rusenova et al. [10] compared phenotypic and genotypic methods in their evaluation of AMR among bovine mastitis isolates of *S. aureus* in Bulgaria. The discrepancies detected for some isolates are concerning and, as recommended by the authors, highlight the need for isolates to be thoroughly characterized.

Biofilm production is considered an important virulence factor for *S. aureus* [11] and coagulase-negative staphylococci (CoNS), which both cause bovine mastitis. Lee and Lee [12] showed that the prevalence of multi-drug resistance (MDR) was significantly higher in strong or moderate biofilm-producing CoNS than in those that were weak or non-formers in samples isolated from normal bulk tank milk in Korea. As biofilm production is associated with persistence of infection and antimicrobial therapy may not eliminate infection, the authors advise effective monitoring and sanitation programs to prevent the contamination of equipment with environmental CoNS, thus limiting the opportunities for cows to be infected with these organisms.

The diverse articles contained in this Special Issue constitute a valuable contribution to our understanding of staphylococcal infection in both farm and companion animals.
